# Tangerines Cultivated on Madeira Island—A High Throughput Natural Source of Bioactive Compounds

**DOI:** 10.3390/foods9101470

**Published:** 2020-10-15

**Authors:** José A. Figueira, Priscilla Porto-Figueira, Jorge A. M. Pereira, José S. Câmara

**Affiliations:** 1 CQM – Centro de Química da Madeira, Universidade da Madeira, Campus Universitário da Penteada, 9020-105 Funchal, Portugal; krudstar@gmail.com (J.A.F.); priscilla.figueira@staff.uma.pt (P.P.-F.); jorge.pereira@staff.uma.pt (J.A.M.P.); 2 Faculdade das Ciências Exatas e Engenharia, Universidade da Madeira, Campus Universitário da Penteada, 9020-105 Funchal, Portugal

**Keywords:** tangerine, d-limonene, thymol, headspace solid-phase microextraction (HS-SPME), gas chromatography mass spectrometry (GC-MS)

## Abstract

Tangerines (*Citrus reticulata*) are popular fruits worldwide, being rich in many bioactive metabolites. The *setubalense* variety cultivated on Madeira Island has an intense aroma easily distinguishable from other tangerines, being traditionally used to enrich several foods and beverages. Nonetheless, *setubalense* volatile composition has never been characterized, and we aimed to unveil the bioactive potential of peels and juices of *setubalense* tangerines and compare them with the *murcott* variety grown in Portugal mainland. Using headspace solid-phase microextraction coupled to gas chromatography mass spectrometry (HS-SPME/GC-MS), we identified a total of 128 volatile organic metabolites (VOMs) in the juice and peels, with d-limonene, γ-terpinene, β-myrcene, α- and β-pinene, o-cymene, and terpinolene, the most dominant in both cultivars. In contrast, *setubalense* juices are richer in terpenes, many of them associated with health protection. Discriminant analysis revealed a pool of VOMs, including β-caryophyllene and E-ocimene, with bioactive properties able to differentiate among tangerines according to variety and sample type (peel vs. juice). This is the first report on the volatile composition of *setubalense* tangerines grown on Madeira Island revealing that its pungent aroma is constituted by secondary metabolites with specific aroma notes and health properties. This is strong evidence of the higher nutraceutical value of such fruit for the human diet.

## 1. Introduction

Citrus fruits are one of the world’s major fruit crops, being produced in many countries of the tropical, subtropical, and temperate borderlines [1]. According to the recent data released by the United States Department of Agriculture (USDA), citrus production in 2019/2020 is estimated to be around 92 million metric tons, representing oranges and tangerines 46.1 and 31.6 million tons, respectively [2]. Citrus fruits are very rich in secondary metabolites with high nutraceutical value, such as vitamin C, folate, flavonoids, coumarins, limonoids, terpenoids, and carotenoids [3]. Some of these metabolites, namely, terpenes, are volatile organic compounds (VOMs), giving the fruit a rich, pungent and distinctive aroma [4,5]. For this reason, citrus fruits and by-products have very important applications, spanning the food industry, cosmetics, and medicine [4]. Moreover, citrus VOMs also constitute a valuable tool for the identification and differentiation of cultivars, hybrids and genotypes [1,4,5]. Citrus fruits are a very heterogeneous group, with dozens of citrus species and varieties, being *Citrus reticulata* one of the most popular. The fruits commonly known as mandarins and tangerines are one of its varieties [5]. Tangerines combine the fresh and acidic notes of other citrus fruits like lemon or lime, with a honey-like sweetness, resulting in unique organoleptic properties that make them very appreciated by consumers [5,6,7]. The core aroma of mandarin juice is essentially composed by nine volatiles, namely, limonene (citrus-like), linalool (floral and citrus), α-terpineol (floral), terpinen-4-ol (woody and earthy), nonanal (piney, floral, and citrus), decanal (fatty and musty), α-pinene (pine-like), β-myrcene (musty and wet soil) and carvone (spearmint and car-away) [6,7]. Additionally, thymol and dimethyl anthranilate have been also mentioned as relevant in the aroma of mandarins [5,7,8]. Many of these volatiles identified in citrus fruits, as in many other fruits and food products, exhibit different bioactive properties (antioxidant, antidiabetic, antiproliferative, etc.) and potential health benefits. Consequently, many of these fruits are considered functional foods with an added value. In this context, current consumers have a growing interest in the provenience and authenticity of their food products as a guarantee of their quality. Therefore, the identification of suitable markers to discriminate these products from others that can be very similar is a crucial regulatory requirement for consumers’ confidence. In this context, the development of fast and reliable methods for analysis of volatiles is received much attention. To achieve this, headspace solid phase microextraction (HS-SPME) combined with gas chromatography–mass spectrometry (GC–MS) is a well-established methodology for VOMs’ analysis, retrieving high sensitivity, reproducibility, and robustness [9]. In recent years, HS-SPME/GC-MS strategy has been successfully applied in the characterization of the volatile profile of new fruits [10]. Furthermore, the use of multivariate statistical methods to process the volatomic data, allowed the discrimination between different cultivars, varieties, and species and even between different maturation stages of the different fruits [9,10,11,12].

The main purpose of this study was to establish the volatile profile of the juice and peels of *setubalense* tangerines grown on Madeira Island, assess their authenticity by comparison with the closely related *murcott* variety cultivated in the mainland, Portugal, and explore its potential as a very rich natural source of several important bioactive compounds with differentiated health properties, namely, thymol, in the variety cultivated in Madeira Island. 

## 2. Materials and Methods 

### 2.1. Chemicals and Materials

All standards used for VOMs confirmation (purity higher than 98.5%) and the C_8_–C_20_ n-alkanes mixture, were obtained from Sigma-Aldrich (St. Louis, MO, USA). The carrier gas in the GC system was helium (ultrapure grade, Air Liquide, Algés, Portugal). The SPME holder for manual sampling, the fibers (divinylbenzene/carboxen on polydimethylsiloxane—DBV/CAR/PDMS, 50 μm DVB layer and 30 μm CAR/PDMS layer and 1 cm fiber length), and the clear glass screw cap vials for SPME with PTFE/silica septa (film thickness 1.3 mm) were purchased from Supelco (Bellefonte, PA, USA). 

### 2.2. Tangerine Samples

Tangerine samples of the varieties *setubalense* (grown in Madeira Island) and *murcott* (grown in Portugal mainland) were selected randomly (10 fruits of each variety) from a local market (Madeira Island), as purchased for consumption. The collection was performed at the beginning of winter (December), which is the optimal time for harvesting. After careful visual inspection of the absence of any sign of fruit degradation, peels and the whole juice, obtained from several fruits of the same variety, were stored under N_2_ (g) atmosphere at −80 °C until analysis. A representative aliquot of each type of sample, 250 mg of peel and 5 mL of juice, were used for triplicate analysis.

### 2.3. HS-SPME Procedure 

The extraction procedure was adopted from previous studies carried out in our laboratory [10,11] with minor modifications. Briefly, 5 mL of tangerine juice was placed into 20 mL headspace glass vial containing a magnetic stirring microbar. NaCl 10% (*w*/*v*) was added to the sample matrix to promote the “salting-out” effect to decrease the solubility of volatile metabolites in the water-based phase. Before sealing the vial, 100 μL of 3-octanol (16.4 μg/L) was also added as internal standard (IS). For the peels, 250 mg of sample was placed into 20 mL of extraction tubes, the proportional amount of IS (5 μL of 3-octanol 16.4 μg/L) was added, and the extraction tubes were sealed. HS-SPME extractions were carried out by exposing the SPME fiber to the headspace of the glass vial (placed in the middle of the vial headspace, about 2 cm above the sample) for 40 min at 40 °C. Finally, the fiber was injected into the GC-MS system at 250 °C for 10 min to attain the thermal desorption of the extracted VOCs. Before each run, blank samples were carried out to ensure any carry-over from the previous analysis. All the experiments were performed in triplicate (*n* = 3) under constant stirring (800 rpm) to improve the extraction. The SPME fibers were thermally conditioned in the GC injector, according to the producer’s recommendations, and also daily for 10 min before the first extraction.

### 2.4. Gas Chromatography–Mass Spectrometry Analysis (GC–MS)

The analysis was carried out on an Agilent 6890N gas chromatograph system (Agilent Technologies, Palo Alto, CA, USA), equipped with a BP-20 polar column, and coupled to an Agilent 5975 quadrupole inert mass selective detector. The full settings and procedure were described previously by Figueira, Porto-Figueira, Pereira, and Câmara [11]. Briefly, trapped VOMs were loaded in the GC-MS using a 6-min splitless injection using He (1.0 mL/min) and separated in BP20-fused silica using a temperature gradient starting at 45 °C (held for 2 min), followed by a temperature gradient (2 °C/min) up to 90 °C, held 3 min, then another increase (3 °C/min) up to 160 °C, held for 6 min, and finally from 160 to 220 °C (6 °C/min) and held at 220 °C for 15 min. The resulting chromatograms were processed using the Enhanced ChemStation software for GC-qMS (Agilent Technologies, Palo Alto, CA, USA). The identification of the VOMs involved the comparison between the GC retention times (RT) of the chromatographic peaks with those of authentic standards, when available, under the same conditions, as well as by the determination of the KI of each identified volatile using a C_8_–C_20_ n-alkanes series solution. Each sample was analyzed in triplicate.

### 2.5. Multivariate Statistical Analysis 

The volatomic data generated was processed with the MetaboAnalyst 4.0 web-based tool [13] (Xia Lab, McGill University, Montreal, QC, Canada) and according to Figueira, Porto-Figueira, Pereira, and Câmara [11]. Briefly, the raw data were normalized (IS ratio correction, sample median, data transformation by cubic root and data scaling by autoscaling). Then, variance (ANOVA, *p* < 0.05) and partial least square discriminant analysis (PLS-DA) were used for variable reduction. PLS-DA reduces the size of the data matrix by eliminating redundant variables (VOMs), thus defining the set of volatiles which define the best separation among the different groups analyzed. Ward’s linkage algorithm and Euclidean distance analysis were used to calculate the ratio of the VOMs, and the resulting metabolic alterations (top VOMs with VIP (variable importance in projection) > 1) were subjected to used HCA analysis to depict distinct clustering patterns among the studied groups. 

## 3. Results and Discussion

### 3.1. Volatomic Profile of Tangerines

**Figure 1:**
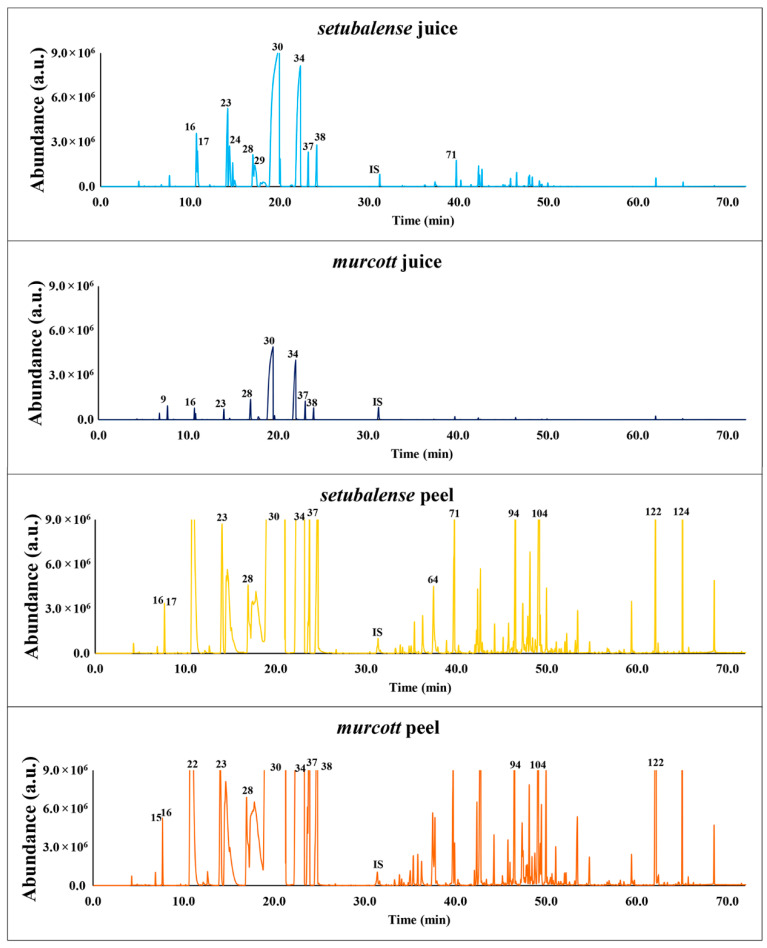
Representative chromatograms of the analyzed samples. Numbers above the peaks refer to the volatile organic metabolites (VOMs) identified in Supplementary Table S1. Peak number identification: 9—ethanol, 15—1-penten-3-one, 16—α-pinene, 17—α-thujene, 22—hexanal, 23—β-pinene (isomer 1), 24—β-pinene (isomer 2), 28—β-myrcene, 29—α-terpinene, 30—d-limonene, 34—γ-terpinene, 37—o-cymene, 38—terpinolene, IS—internal standard (3-octanol), 64—decanal, 71—linalool, 94—α-terpineol, 104—α-farnesene, 122—dimethyl anthranilate, 124—thymol.

The volatile profiles from the two tangerines varieties analyzed are quite different, being the *setubalense* juice clearly richer than *murcott* in terms of number and intensity of VOMs characterized (Figure 1). Regarding the peels, we observe that their volatile composition is much richer (number and intensity of VOMs) than the juices, while minor differences have been observed between varieties (Figure 1). Overall, 128 different VOMs have been identified, from which 109 were in the *murcott* and 117 were in the *setubalense* variety. The detailed list of all VOMs identified and respective experimental data, including the retention time and relative peak area is available in Supplementary Table S1. The contribution of each VOM for the total volatile fraction expressed as relative peak area was calculated according to Equation (1):

(1) Peak area of analytePeak area of internal standard.

**Figure 2:**
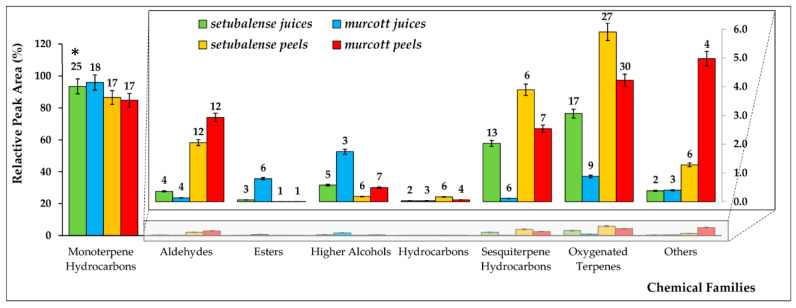
VOMs identified in juices and peels of *setubalense* and *murcott* tangerines, by chemical family. The numbers close the bars (*) indicates the number of identified VOMs in each chemical family.

During sample preparation, we observed a stronger and distinctive smell for the *setubalense* tangerines. This agrees with the fact that the sum of the relative peak areas of the 75 VOMs identified in *setubalense* juice is 5 times higher than the 56 VOMs identified in *murcott* juices. In contrast, the sum of the relative peak areas obtained for the 89 VOMs identified in *setubalense* peels is only 20% higher than found for the 91 VOMs identified in *murcott* peels samples (Supplementary Table S1). Monoterpenes hydrocarbons are by far the functional class more abundant in all samples analyzed, representing over 93% and 84% of the volatile fractions of tangerine juices and peels, respectively (Figure 2). Such abundance is mainly due to d-limonene, followed by γ-terpinene, β-myrcene, β-pinene, o-cymene, α-pinene, and terpinolene, which are the most abundant VOMs identified in all tangerine samples (Supplementary Table S1). This suggests that the VOMs with lower abundance should have an important contribution for the distinct profile of *setubalense* juices and peels in comparison with *murcott*. Further olfactometry analysis would be very interesting to clarify this question.

#### 3.1.1. Volatomic Profile of Tangerine Juices

**Figure 3:**
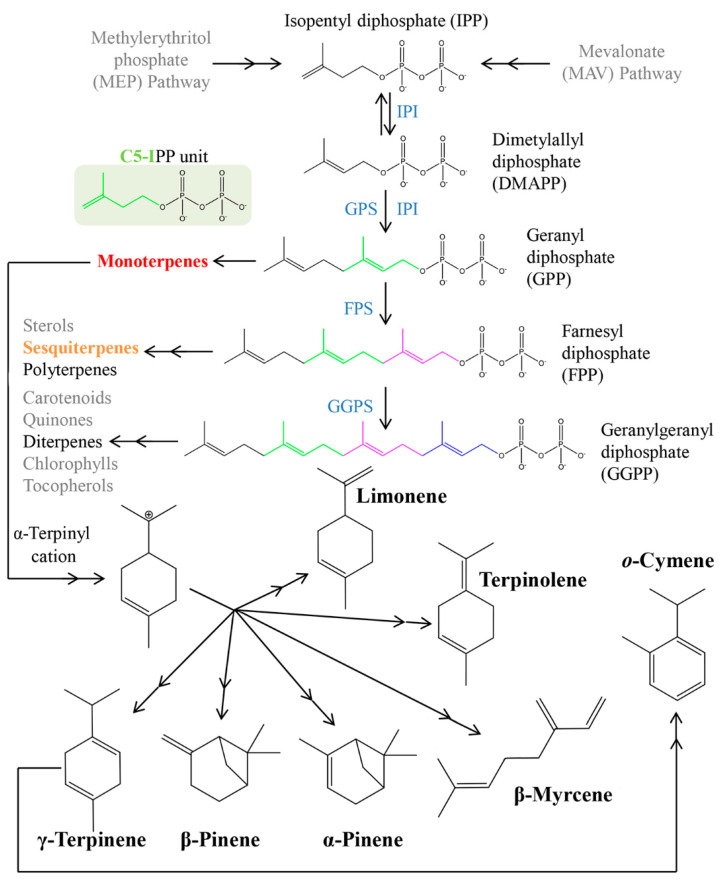
Main reactions cascade involved in the formation of the most abundant terpenoids identified in this work (adapted from [15]).

As can be observed in Figure 2, sesquiterpene hydrocarbons and oxygenated terpenes are clearly more abundant in *setubalense* juices, both in terms of the number of VOMs identified, as in relative peak areas for each family of compounds. In contrast, the relative peak areas for higher alcohols and esters are much higher in *murcott*. Ethyl acetate, for instance, is three times more abundant in *murcott* comparatively to *setubalense* juices. Beyond the relative peak areas, 28 VOMs were exclusively identified in the *setubalense* variety juices (7 monoterpenes, 8 sesquiterpenes, and 10 terpenoids; Supplementary Table S1). Since terpenes result from the Methylerythritol 4-phosphate (MEP) and the mevalonate (MAV) pathways [14,15] (Figure 3), this result suggests a lower activity of these pathways in the *murcott* relative to the *setubalense* variety, particularly in the cytosol where the final steps of sesquiterpenes biosynthesis occur. In opposition, ethyl isobutyrate, propyl acetate, and ethyl 2-butenoate were exclusively identified in *murcott* juices. The closely related mandarin juice contains nine volatiles that are considered as core aromatic volatiles, namely, linalool, α-terpineol, terpinen-4-ol, nonanal, decanal, carvone, limonene, α-pinene, and β-myrcene [6,7]. All these VOMs were identified in the tangerine samples analyzed in this work. Beyond this, more VOMs, namely, terpenes, were identified in this study in comparison to a recent report [16].

#### 3.1.2. Volatomic Profile of Tangerine Peels

When compared to the juice samples, the volatomic profile of tangerine peels is more complex, being the sum of relative peak areas 50 times higher in the *murcott* variety and 1.25 times higher in the *setubalense* variety than the respective juice samples. Regarding the number of VOMs, there was also an increment in the number of VOMs identified in the peels. Overall, 89 VOMs were identified in *murcott* peels (56 in the juices), whereas in *setubalense* variety, the variation was lower (87 vs. 75 VOMs in peels and juices, respectively; Supplementary Table S1). As shown in Figure 2, these differences are mainly due to aldehydes and oxygenated terpenes, whose number of VOMs triplicates from the juices to the peels. 

The variation in the number of oxygenated terpenes found in the peel samples, when compared to juices samples, can be easily explained by the interaction with the atmospheric air that favors the oxidation of many metabolites, including VOMs. Similarly, 3 ketones and 3 furans, grouped as “others” in Figure 2, exhibit a robust predominance in the volatile composition of the peels. Regarding this, 1-penten-3-one accounts for half of the relative peak area of the “others” family of the tangerine peel samples from the *murcott* variety. In contrast, esters almost disappear, being only ethyl acetate identified in the peels of both varieties. Overall, 33 more VOMs were identified in the *murcott* variety and more 12 VOMs in the *setubalense* variety. 

### 3.2. Tangerines Bioactive Potential

Many of the compounds identified in *setubalense* and *murcott* tangerines have been previously reported with a myriad of bioactivities. This includes antioxidant [17,18,19,20], anti-inflammatory [17], antidiabetic [19,21], antileishmanial [22,23], antimicrobial [19,22,24,25], cytotoxic [22,23], antitumor [26], and antiproliferative activities [27,28]. Furthermore, there are evidence pointing to other protective effects against Alzheimer’s [29] and tuberculosis [30]. Most of these bioactive VOMs are terpenes that, as already referred, can be obtained from the MEP and MAV pathways through the isopentyl diphosphate (IPP) precursor [14,15]. Figure 3 shows a simplified diagram of the reaction cascade producing the most abundant terpenes identified in the *setubalense* and *murcott* tangerines analyzed in this work.

Overall, bioactivity is recognized not only to the major VOMs identified in the tangerines but also to other present in lower abundance, as thymol and dimethyl anthranilate, whose benefits to human health are widely known [31,32] (Table 1). Thymol and dimethyl anthranilate have also been reported as having an important contribution to the mandarin aroma, being used in synthetic tangerine flavour [7,8]. 

According to Ladanyia and Ladanyia [33], the distinctive notes in the aroma of the Mediterranean mandarin oil were due this two VOMs, accounting dimethyl anthranilate to 0.85% and thymol to 0.08% of the volatomic profile. In this work, dimethyl anthranilate represents 1.18% and 2.35% and thymol represents 0.87% and 0.58% of the volatile profile of the peels of *setubalense* and *murcott* varieties, respectively. Such abundance in thymol and dimethyl anthranilate supports the use of *setubalense* tangerine as a potential and interesting alternative to the thyme oils, which are often associated to some allergic reactions, even when it is used diluted [32]. Another interesting VOM identified in this work is cosmene. This compound represents almost 20% of the oil obtained from the roots of *Eupatorium adenophorum* Spreng (Asteraceae) [34]. This native plant from Central America, commonly known as Mexican devil became invasive worldwide, being used in the folk medicine for its bioactive properties (antimicrobial, antiseptic, etc. [34]). In fact, Ahluwalia, Sisodia, Walia, Sati, Kumar, and Kundu [34] reported antimicrobial, antioxidant, and phytotoxic properties for the root’s oils of this plant and such effect will be certainly elicit by cosmene given its high abundance in the roots extracts.

### 3.3. Classification of Tangerine Samples

**Figure 4:**
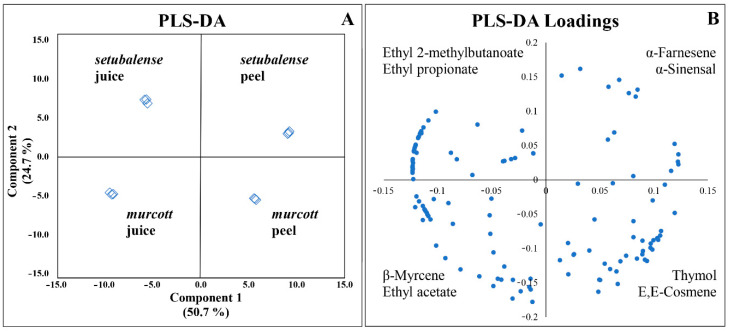
Multivariate statistical analysis (MVSA) using partial least square discriminant analysis (PLS-DA) of the volatomic data obtained (**A**) and identification of the variables (VOMs) responsible for the differentiation of the samples (**B**).

In order to evaluate the potential of the volatile fingerprints obtained and discriminate tangerine samples according to the variety and sample type (juice vs. peel), a statistical analysis of the volatomic data matrix was performed using MetaboAnalyst 4.0 web-based tool [13]. The complexity of the data was reduced through normalization, as described in the experimental section. Then univariate statistical analysis (ANOVA, *p* ≤ 0.05) was carried out to verify the significant statistical differences among the concentrations of VOMs in each sample group (*p* < 0.05; Supplementary Table S2). Multivariate statistical analysis (MVSA) using PLS-DA was then performed to assess if there were significant VOMs signatures between each group. The results obtained show four different clusters discriminating each of the groups under study (Figure 4).

As Figure 4A shows, the first component explains 50.7% of the variance and separate the tangerine “juice” from “peel”. The second component contributes for 24.7% of the total variance of the model and separate the tangerine variety *murcott* from *setubalense*. Figure 4B presents the variables that are most contributed for the differentiation of the tangerines by variety and type of sample. Accordingly, ethyl 2-methyl butanoate, ethyl propionate, and 2-methyl-furan are more associated to *setubalense* juice; ethyl acetate, β-myrcene, and β-phellandrene to *murcott* juice; α-farnesene, α-sinensal, and camphor more associated to *setubalense* peel; and cis-β-terpineol, cosmene, and thymol to *murcott* peel. 

**Figure 5:**
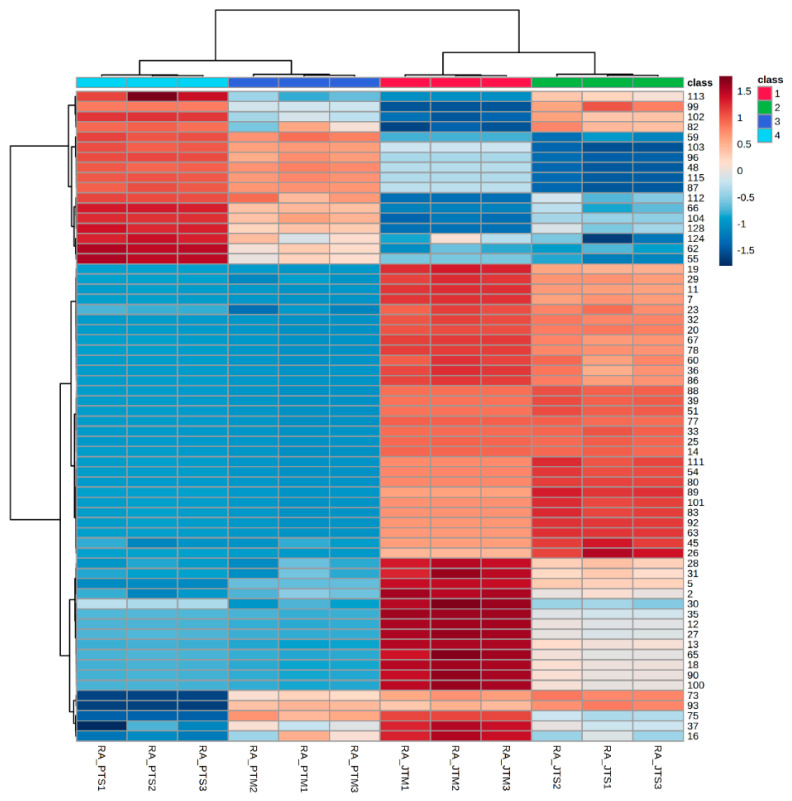
Hierarchical cluster analysis (HCA) analysis of the volatomic data obtained in this work, as described in Materials and Methods section.

Hierarchical cluster analysis (HCA) was also performed using the 64 most significant VOMs identified in tangerine samples, as described in Section 2.5. This strategy allows a better identification of the inherent clustering patterns between each variety, in complementarity with the statistical analysis carried out previously. The result of this treatment can be visualized in the heatmap plot (Figure 5). 

Overall, the set of variables that contributed to the differentiation of the tangerines by variety have the potential to be used for product identification and authenticity. Furthermore, tangerines of the *setubalense* variety contain higher amounts of bioactive analytes than *murcott* tangerines (Table 1). Therefore, this bioactive signature supports higher health benefits for the *setubalense* tangerines grown in Madeira Island, as can be used to the discrimination of the two varieties analyzed.

## 4. Conclusions

In this study, HS-SPME-GC/MS was used to characterize for the first time the volatile composition of a tangerine variety cultivated in Madeira Island (*setubalense*) and compare it with the *murcott* variety grown in mainland. This comparison involved the juice and peels of both varieties and allowed the identification of 128 VOMs, 107 VOMs in *murcott,* and 115 VOMs in *setubalense* variety, which is considerably higher than most of the previous reports using the same methodology to analyze different tangerine varieties. To the best of our knowledge, only the tangerine hybrid *9-4 × Blood4x* analyzed by Miyazaki, Plotto, Goodner, and Gmitter [5] presented a similar number of VOMs (118 VOMs) than the *setubalense* variety. The volatile composition of the peels is more complex than the juices, and the *setubalense* distinctive aroma corresponds to a higher abundance of common VOMs, as most of them have been previously reported with important bioactive properties. d-limonene, γ-terpinene, β-myrcene, β-pinene, o-cymene, α-pinene, and terpinolene are among the most abundant VOMs, but *setubalense* tangerines are also very rich in thymol, making this fruit a very interesting alternative to thyme oils.

The uniqueness of *setubalense* tangerines was shown through a statistical analysis that differentiate this variety from the common *murcott* grown in Portugal mainland. Accordingly, ethyl 2-butenoate, (E)-2-decenal, cycloheptane, and carvone associated to the *murcott* variety and α-himachalene, β-santalene, α-selinene, and α-sinensal associated to the *setubalense* variety, were the VOMs that most contributed to the differentiation of two tangerine varieties.

## Figures and Tables

**Table 1:** Potential bioactive effects of the most important volatile organic metabolites (VOMs) identified in this study.

		Juice		Peels		VOMs Potential Bioactive Effects ^3^										
**PN ^1^**	**VOMs**	***Set*/*Mur*** **Ratio ^2^**	***T* Test (*p* < 0.05)**	***Set*/*Mur* Ratio ^2^**	***T* Test (*p* < 0.05)**	Antibacterial	Antidepressant	Antidiabetic	Anti-inflammatory	Antileishmanial	Antifungal	Antioxidant	Antiproliferative	Antitumor	Cytotoxic	**References**
16	α-pinene	8.50	0.0218	0.40	0.0367			ǂ		ǂ	ǂ	ǂ	ǂ	ǂ	ǂ	[18,21,22,23,24,26,27,28]
17	α-thujene	10.0	0.0135	39.8	0.0002	ǂ					ǂ	ǂ		ǂ		[35,36]
21	camphene	15.8	0.0079	0.10	0.0025	ǂ						ǂ	ǂ			[18,24,28]
23	β-pinene	17.5	0.0182	1.00	0.3719						ǂ	ǂ	ǂ	ǂ		[18,24,26,28]
25	sabinene	23.3	0.0115	–	–	ǂ				ǂ				ǂ	ǂ	[22,24,26]
28	β-myrcene	5.60	0.0054	0.70	0.3413			ǂ	ǂ	ǂ	ǂ	ǂ	ǂ	ǂ	ǂ	[17,19,22,24,26,28]
29	α-terpinene	7.50	0.0076	0.90	0.0854	ǂ				ǂ	ǂ			ǂ	ǂ	[22,24,26]
30	limonene (isomer)	4.40	0.0086	1.20	0.0979	ǂ		ǂ	ǂ		ǂ	ǂ	ǂ	ǂ	ǂ	[18,19,20,21,22,23,24,25,26,27,28]
31	β-phellandrene	7.90	0.0201	0.70	0.0827	ǂ		ǂ		ǂ		ǂ			ǂ	[19,22,26]
33	(E)-ocimene	31.3	0.0052	–	–	ǂ		ǂ	ǂ			ǂ				[17,19]
34	γ-terpinene	4.90	0.0001	2.10	0.0283						ǂ	ǂ		ǂ		[18,24,26]
37	o-cymene	3.00	0.0033	0.20	0.0015	ǂ			ǂ		ǂ	ǂ			ǂ	[35,37,38]
38	terpinolene	6.00	0.0001	1.60	0.0013	ǂ								ǂ		[24,26]
53	*p*-cymenene	7.00	0.0004	1.90	0.2046	ǂ					ǂ	ǂ			ǂ	[37,38]
55	Cosmene ^4^	–	–	3.40	0.0000	ǂ					ǂ	ǂ				[34]
71	linalool	17.3	0.0000	1.90	0.0000	ǂ		ǂ	ǂ			ǂ	ǂ			[17,20,24,25,28]
79	thymol methyl ether	8.34	0.0004	3.00	0.0000	ǂ			ǂ		ǂ	ǂ	ǂ			[31,32]
82	β-caryophyllene	62.5	0.0009	1.20	0.0000	ǂ		ǂ		ǂ		ǂ	ǂ		ǂ	[20,22,23,24,27,28]
94	α-terpineol	13.7	0.0010	1.60	0.0030	ǂ				ǂ	ǂ	ǂ		ǂ	ǂ	[18,23,24,25,26]
107	perilla aldehyde	9.40	0.0017	1.40	0.0164	ǂ	ǂ		ǂ			ǂ				[39]
122	dimethyl anthranilate	4.94	0.0105	0.6	0.0012	ǂ					ǂ					[40]
124	thymol	11.5	0.0073	1.9	0.0004	ǂ			ǂ		ǂ	ǂ				[31,32]

Legend: ^1^ VOMs peak number in the chromatograms (Figure 1); ^2^ ratio between the relative peak areas obtained for *setubalense* and *murcott* tangerines; ^3^ potential bioactive effect indicates the type of bioactive effects reported for each of the VOMs referred in the table, *mur*—*murcott* cultivar, *set—setubalense* cultivar; ^4^ strong evidence for the indicated bioactivity, ǂ reported bioactive effect.

## Supplementary Materials

The following are available online at https://www.mdpi.com/2304-8158/9/10/1470/s1, Supplementary Table S1: Volatile organic metabolites (VOMs) identified in tangerines juices and peels from the *setubalense* and *murcott* varieties; Supplementary Table S2: VOM identified by One-way ANOVA and post hoc analysis, found as statistically significant for *p* < 0.05.

## Abbreviations

EI	electron: impact mode
CAR	carboxen
DBV	divinylbenzene
GC-MS	gas chromatography-mass spectrometry
HCA	hierarchical cluster analysis
HS-SPME	head space solid phase microextraction
IS	internal standard
MVSA	multivariate statistical analysis
NIST	National Institute of Standards and Technology
PDMS	polydimethylsiloxane
PLS-DA	partial least square discriminant analysis
PTFE	polytetrafluoroethylene
RT	retention time
VIP	variable importance in projection
VOMs	volatile organic metabolites
