# Supplementary material for: Tangerines Cultivated on Madeira Island—A High Throughput Natural Source of Bioactive Compounds
Source: Foods. 2020 Oct 15;9(10):1470. doi: 10.3390/foods9101470 (PMC7602526; doi:10.3390/foods9101470)
Supplement: Supplementary file 1 [file foods-09-01470-s001.pdf]

Supplementary Table S1

Volatile organic metabolites (VOMs) identified in juices and peels of tangerines from *setubalense* and *marcott* varieties.

	VOMs (by chemical family)	Cas #	RT (min) [a]	RI_{calc} [b]	RI_{lit} [c]	Relative Peak Area[d] (RSD<10%)			
						Juice		Peel	
						murcott	***setubalense***	***murcott***	***setubalense***
**Aldehydes**									
**2**[e]	**Acetaldehyde**	75-07-0	4.90	716	725	1.18	2.33	12.46	5.82
**22**	**Hexanal**	66-25-1	12.70	1045	1045	0.42	2.67	3626.29	122.31
**40**	Octanal	124-13-0	25.09	1252	1268	nd[f]	nd	95.49	14.04
**48**	**Nonanal**	124-19-6	31.41	1345	1343	nd	nd	290.35	423.45
**64**	**Decanal**	112-31-2	37.40	1447	1446	3.26	66.26	804.32	2402.25
**67**	**Benzaldehyde**	100-52-7	38.20	1462	1471	0.13	0.66	nd	nd
**69**	(E)-2-Nonenal	18829-56-6	39.00	1476	1500	0.14	nd	7.68	25.20
**70**	cis-4-Decenal	21662-09-9	39.42	1484	1523	nd	nd	5.86	14.64
**87**	(E)-2-Decenal	3913-81-3	44.22	1584	1597	nd	nd	200.17	384.60
**96**	**Dodecanal**	112-54-9	47.36	1656	1685	nd	nd	317.24	914.88
**115**	2-Dodecenal	4826-62-4	53.41	1800	1830	nd	nd	324.62	635.96
**116**	(E,Z)-2,6-nonadienal	557-48-2	54.75	1829	1601	nd	nd	96.76	167.40
**117**	Tetradecanal	124-25-4	56.69	1869	1911	nd	nd	22.76	39.36
	Σ[g] =13			**Total Relative Area** [h]		5.13	71.91	5803.99	5149.89
**Esters**									
**5**	**Ethyl acetate**	141-78-6	6.80	849	856	26.17	8.90	4.72	0.87
**11**	**Ethyl propionate**	105-37-3	8.40	925	931	1.41	2.57	nd	nd
**12**	**Ethyl isobutyrate**	97-62-1	8.60	932	954	0.78	nd	nd	nd
**13**	**Propyl acetate**	109-60-4	8.80	939	962	0.14	nd	nd	nd
**19**	Ethyl 2-methylbutanoate	7452-79-1	11.50	1018	1015	1.21	1.25	nd	nd
**27**	Ethyl 2-butenoate	10544-63-5	16.70	1121	1122	1.18	nd	nd	nd
	Σ=6			**Total Relative Area**		30.89	12.72	4.72	0.87
**Higher Alcohols**									
**6**	**Methanol**	67-56-1	6.89	854	860	nd	nd	147.18	76.14
**8**	**Isopropyl Alcohol**	67-63-0	7.50	888	884	nd	nd	725.84	nd
**9**	**Ethanol**	64-17-5	7.60	893	899	65.68	65.71	2.72	355.93
**41**	(Z)-4-Hexen-1-ol	543-49-7	26.35	1269	1280	nd	nd	nd	6.75

**44**	**1-Hexanol**	111-27-3	28.46	1296	1294	nd	nd	7.74	1.09
**46**	3-Hexen-1-ol	544-12-7	30.40	1328	1327	nd	0.78	18.44	9.03
**73**	**1-Octanol**	111-87-5	40.20	1498	1494	1.12	44.42	65.38	nd
**90**	**1-Nonanol**	143-08-8	45.00	1600	1639	0.29	nd	nd	nd
**111**	(E)-2-Nonen-1-ol	31502-14-4	51.30	1750	1722	nd	4.01	nd	nd
	Σ=10			**Total**	**Relative Area**	67.09	114.93	967.30	448.94
**Hydrocarbons**									
**1**	**1,4-pentadiene**	591-93-5	4.65	695	646	nd	nd	0.88	2.20
**36**	1-Dodecene	112-41-4	22.30	1211	1232	1.05	3.39	nd	nd
**42**	**Tridecane**	629-50-5	26.40	1270	1273	nd	0.73	7.83	79.67
**43**	(E)-6-Tridecene	6434-76-0	28.23	1293	nd	nd	nd	nd	6.32
**49**	(3E,5Z)-1,3,5-Undecatriene	51447-08-6	31.65	1348	1382	nd	nd	68.74	71.39
**52**	**Tetradecane**	629-59-4	33.30	1374	1375	nd	nd	36.36	104.59
**60**	**1-Tetradecene**	1120-36-1	35.40	1408	1429	0.30	1.94	nd	nd
**68**	**Pentadecane**	629-62-9	38.94	1475	nd	nd	nd	13.35	135.34
**84**	**Hexadecane**	544-76-3	43.73	1574	nd	nd	nd	3.88	6.97
**119**	3-propyl-Cyclohexene	3983-06-0	60.69	1968	nd	nd	nd	nd	11.31
	Σ=10			**Total**	**Relative Area**	1.35	6.06	131.04	417.78
**Monoterpene Hydrocarbons**									
**14**	Tricyclene	508-32-7	10.20	984	995	nd	1.08	nd	nd
**16**	**α-Pinene**	7785-70-8	10.70	998	1001	50.49	429.86	8108.73	3108.77
**17**	α-Thujene	2867-05-2	10.80	1001	1003	24.03	240.76	78.23	3114.79
**20**	**α-Fenchene**	471-84-1	11.90	1027	1027	0.18	1.69	nd	nd
**21**	**Camphene**	79-92-5	12.19	1034	1034	0.88	13.91	620.88	31.74
23	β-Pinene (isomer 1)	127-91-3	14.10	1073	1078	52.67	921.27	2706.30	2832.13
**24**	β-Pinene (isomer 2)	18172-67-3	14.40	1079		nd	368.04	nd	nd
**25**	**Sabinene**	3387-41-5	14.79	1086	1086	6.29	146.39	nd	nd
**26**	β-Thujene	28634-89-1	14.90	1088	1107	nd	51.44	nd	nd
**28**	**β-Myrcene**	123-35-3	17.00	1127	1127	142.08	794.50	2964.61	2064.57
**29**	**α-Terpinene**	99-86-5	17.80	1141	1141	28.58	213.43	109.88	102.70
**30**	**D-Limonene**	5989-27-5	19.50	1169	1176	2346.61	10337.65	113983.25	134417.14
**31**	**β-Phellandrene**	555-10-2	19.70	1172	1177	13.85	109.10	704.76	523.59
**32**	(Bis(1-methylethylidene)-cyclobutane	3642-14-6	19.90	1175	1460	0.33	2.46	nd	nd
**33**	**(E)-Ocimene**	3779-61-1	21.10	1192	1196	0.91	28.44	nd	nd
**34**	**γ-Terpinene**	99-85-4	21.80	1203	1210	877.32	4327.34	29060.28	61526.81
**35**	**Z-Ocimene**	3338-55-4	22.10	1208	1210	3.26	nd	nd	nd
**37**	**o-Cymene**	527-84-4	23.00	1221	1225	93.05	276.77	4501.03	929.45

**38**	**Terpinolene**	586-62-9	24.00	1236	1239	61.99	373.69	4944.98	7887.33
**39**	2-Carene	554-61-0	24.30	1241	1273	nd	1.69	nd	nd
**45**	(4E,6Z)-allo-Ocimene	7216-56-0	30.00	1321	1327	nd	1.91	3.90	2.87
**53**	p-Cymenene	1195-32-0	33.80	1382	1383	1.02	7.19	77.78	151.49
**54**	1,3,8-p-Menthatriene	21195-59-5	33.90	1383	1387	nd	3.32	nd	nd
**55**	Cosmene	460-01-5	34.20	1388	1460	nd	0.66	36.15	123.76
**91**	2,6-Dimethyl-2,6-octadiene	2792-39-4	45.20	1604	1604	0.17	9.92	26.10	179.54
	Σ=25			**Total**	**Relative Area**	3703.72	18662.53	167926.86	216996.67
**Sesquiterpene Hydrocarbons**									
**61**	α-Cubebene	17699-14-8	35.70	1414	1435	0.16	0.33	109.55	27.20
**63**	δ-Elemene	20307-84-0	36.25	1425	1465	nd	7.39	nd	nd
**65**	Copaene	3856-25-5	37.50	1449	1454	0.54	nd	nd	nd
**72**	β-Cubenene	13744-15-5	39.85	1492	1519	nd	nd	137.26	
**77**	α-Santalene	512-61-8	41.50	1526	1526	nd	0.96	nd	nd
**78**	Allo-Aromadendrene	25246-27-9	41.60	1528	1618	nd	0.34	nd	nd
**80**	α-Bergamotene	17699-05-7	42.20	1541	1542	0.97	147.31	nd	nd
**82**	**β-Caryophyllene**	87-44-5	42.60	1550	1548	1.94	121.19	1460.97	1766.45
**83**	**α-Himachalene**	3853-83-6	43.40	1567	1566	nd	5.84	nd	nd
**88**	Elixene	3242-08-8	44.40	1587	1514	nd	1.49	nd	nd
**89**	β-Santalene	511-59-1	44.90	1598	1599	nd	12.36	nd	nd
**92**	(Z)-β-Farnesene	28973-97-9	45.60	1614	1613	nd	6.57	nd	nd
**101**	α-Selinene	473-13-2	48.00	1671	1719	nd	5.40	nd	nd
**102**	β-Bisabolene	495-61-4	48.20	1676	1723	0.37	60.69	426.24	1672.20
**103**	γ-Elemene	30824-67-0	48.47	1682	1651	nd	nd	119.02	239.37
**104**	α-Farnesene	502-61-4	49.00	1694	1744	0.41	35.23	2383.36	5960.78
**106**	-δ-Cadinene	483-76-1	49.45	1705	1716	nd	nd	385.27	95.52
	Σ=17			**Total**	**Relative Area**	4.40	405.09	5021.67	9761.52
**Oxygenated Terpenes**									
**50**	Perillene	539-52-6	32.97	1369	1369	nd	nd	2.21	2.14
**56**	**cis-Limonene oxide**	4680-24-4	34.33	1390	1388	nd	nd	11.50	16.47
**57**	**trans-Limonene oxide**	6909-30-4	35.04	1401	1398	nd	nd	199.31	168.22
**59**	cis-β-Terpineol	7299-41-4	35.30	1406	1404	nd	0.58	322.93	422.05
**62**	Citronellal	106-23-0	36.20	1424	1457	0.40	8.57	360.03	1034.77
**66**	**Camphor**	76-22-2	37.90	1456	1455	nd	1.77	66.47	119.87
**71**	**Linalool**	78-70-6	39.60	1487	1486	10.52	182.49	1559.91	2959.94
**74**	(E)-p-2-Menthen-1-ol	29803-81-4	40.45	1503	1552	nd	nd	1.83	2.05
**76**	Isopulegol	89-79-2	40.82	1511	1565	nd	nd	5.01	9.67

**81**	**Terpinen-4-ol**	562-74-3	42.30	1543	1585	6.77	82.96	523.22	883.81
**85**	Umbellulone	24545-81-1	43.93	1578	1610	nd	nd	14.04	nd
**93**	**α-Caryophyllene**	106-26-3	45.80	1619	1641	0.55	45.42	181.72	nd
**94**	**α-Terpineol**	98-55-5	46.40	1633	1633	7.71	105.29	1951.38	3213.62
**95**	**Borneol**	507-70-0	46.70	1641	1638	nd	nd	18.52	75.25
**97**	**Piperitone**	89-81-6	47.66	1663	1662	nd	nd	56.80	79.86
**98**	Neryl propionate	105-91-9	47.80	1667	1758	nd	45.99	76.06	160.10
**99**	Geranial	141-27-5	47.88	1668	1714	nd	49.01	141.05	553.47
**100**	**Carvone**	2244-16-8	47.89	1668	1715	0.34	nd	nd	nd
**105**	**(R)-Citronellol**	1117-61-9	49.30	1701	1699	2.28	16.33	305.96	455.00
**107**	**Perilla aldehyde**	2111-75-3	49.90	1716	1716	2.89	27.13	636.71	921.07
**108**	**γ-Isogeraniol**	13066-51-8	50.12	1721	1800	nd	nd	55.34	nd
**109**	**Nerol**	106-25-2	50.60	1733	1731	nd	2.97	87.03	33.62
**110**	(E,E)-2,4-Decadienal	25152-84-5	51.04	1744	1771	nd	nd	116.69	144.06
**112**	**cis-Carveol**	1197-06-4	52.00	1767	1832	nd	2.58	127.82	154.01
**113**	**Cherry propanol**	1197-01-9	52.60	1781	1840	nd	2.06	20.20	45.33
**114**	**Carveol**	99-48-9	53.20	1795	1845	nd	4.17	14.77	176.11
**118**	**Perillyl alcohol**	536-59-4	59.69	1940	1959	nd	nd	25.02	35.15
**120**	**Nerolidol**	7212-44-4	61.06	1978	1990	nd	nd	nd	9.33
**123**	**Eugenol**	97-53-0	64.58	2069	2117	nd	nd	48.28	nd
**124**	**Thymol**	89-83-8	65.00	2080	2166	2.49	28.63	1143.30	2190.10
**125**	4-Isopropyl-3-methylphenol	3228-02-2	65.68	2097		nd	nd	72.95	50.82
**128**	α-Sinensal	17909-77-2	68.50	2166	2271	nd	4.95	219.08	878.83
	Σ=32			**Total**	**Relative Area**	33.94	610.90	8365.13	14794.71
**Others**									
**3**	Carbon disulfide	75-15-0	5.08	731	745	nd	nd	3.55	1.11
**4**	**Acetone**	67-64-1	5.76	782	775	nd	nd	5.12	1.96
**15**	**1-penten-3-one**	1629-58-9	10.24	985	991	nd	nd	4771.24	4.54
**7**	**2-Methyl-furan**	534-22-5	6.90	854	858	0.42	1.25	nd	nd
**10**	2-Ethyl-furan	3208-16-0	8.26	920	922	nd	nd	12.64	1.48
**18**	**Toluene**	108-88-3	11.10	1008	1008	0.24	nd	nd	nd
**51**	2,3-Dihydro-2-methylbenzofuran	1746-11-8	33.10	1371		nd	1.71	nd	nd
**58**	**Acetic acid**	64-19-7	35.10	1402	1403	nd	0.45	335.74	12.00
**75**	6,6-Dimethyl-2-methylidene-norpinan-3-one	16812-40-1	40.57	1505		nd	nd	0.45	0.75
**79**	**Thymol methyl ether**	1076-56-8	42.00	1537	1563	0.43	3.59	55.95	169.84
**86**	**p-Tolualdehyde**	104-87-0	44.10	1581	1605	0.86	3.12	nd	nd

**121**	**Octanoic acid**	124-07-02	61.75	1996	2021	nd	nd	nd	30.41
**122**	**Dimethyl anthranilate**	85-91-6	62.00	2003	2042	13.19	65.20	4651.75	2970.61
**126**	**Methyl anthranilate**	134-20-3	66.20	2110	2198	0.18	nd	10.62	4.78
**127**	**n-Decanoic acid**	334-48-5	67.16	2134	2229	nd	nd	6.10	6.57
**129**	**Indole**	120-72-9	71.58	2238	2398	nd	nd	3.11	10.80
	Σ=16				**Total relative area**	15.32	75.31	9856.28	3214.85
					**TOTAL RELATIVE PEAK AREA:**	**3961.83**	**20059.46**	**198176.99**	**250885.22**
					**NUMBER OF IDENTIFIED VOMs:**	**56**	**75**	**89**	**87**

[a] RT: retention time expressed in min.

[b] RI_{calc}: experimental Kovat's index.

[c] RI_{lit}: Kovat's index reported in the literature.

[d] Relative Peak Area: (VOM peak area/Internal Standard peak area).

[e] Peak identification number ordered by the VOC retention time.

[f] nd: Not detected

[g] Sum of VOMs within chemical family

[h] Total relative area within chemical family

VOCs indicated in **bold** were confirmed with commercial standards

Supplementary Table S2: VOM identified by One-way ANOVA and post-hoc analysis, found as statistically significant for ($p < 0.05$).

VOM	chi.squared	p.value	=-LOG10(p)	FDR	Post-Hoc
3	10.532	0.014546	1.8373	0.022136	NA
4	10.532	0.014546	1.8373	0.022136	NA
6	10.532	0.014546	1.8373	0.022136	NA
8	10.532	0.014546	1.8373	0.022136	NA
10	10.532	0.014546	1.8373	0.022136	NA
15	10.532	0.014546	1.8373	0.022136	NA
24	10.532	0.014546	1.8373	0.022136	NA
26	10.532	0.014546	1.8373	0.022136	NA
39	10.532	0.014546	1.8373	0.022136	NA
41	10.532	0.014546	1.8373	0.022136	NA
43	10.532	0.014546	1.8373	0.022136	NA
44	10.532	0.014546	1.8373	0.022136	NA
48	10.532	0.014546	1.8373	0.022136	NA
50	10.532	0.014546	1.8373	0.022136	NA
51	10.532	0.014546	1.8373	0.022136	NA
52	10.532	0.014546	1.8373	0.022136	NA
54	10.532	0.014546	1.8373	0.022136	NA
55	10.532	0.014546	1.8373	0.022136	NA
56	10.532	0.014546	1.8373	0.022136	NA
59	10.532	0.014546	1.8373	0.022136	NA
63	10.532	0.014546	1.8373	0.022136	NA
66	10.532	0.014546	1.8373	0.022136	NA
68	10.532	0.014546	1.8373	0.022136	NA
70	10.532	0.014546	1.8373	0.022136	NA

72	10.532	0.014546	1.8373	0.022136	NA
74	10.532	0.014546	1.8373	0.022136	NA
75	10.532	0.014546	1.8373	0.022136	NA
76	10.532	0.014546	1.8373	0.022136	NA
78	10.532	0.014546	1.8373	0.022136	NA
83	10.532	0.014546	1.8373	0.022136	NA
84	10.532	0.014546	1.8373	0.022136	NA
85	10.532	0.014546	1.8373	0.022136	NA
87	10.532	0.014546	1.8373	0.022136	NA
88	10.532	0.014546	1.8373	0.022136	NA
89	10.532	0.014546	1.8373	0.022136	NA
92	10.532	0.014546	1.8373	0.022136	NA
95	10.532	0.014546	1.8373	0.022136	NA
96	10.532	0.014546	1.8373	0.022136	NA
97	10.532	0.014546	1.8373	0.022136	NA
98	10.532	0.014546	1.8373	0.022136	NA
101	10.532	0.014546	1.8373	0.022136	NA
103	10.532	0.014546	1.8373	0.022136	NA
106	10.532	0.014546	1.8373	0.022136	NA
108	10.532	0.014546	1.8373	0.022136	NA
110	10.532	0.014546	1.8373	0.022136	NA
111	10.532	0.014546	1.8373	0.022136	NA
112	10.532	0.014546	1.8373	0.022136	NA
113	10.532	0.014546	1.8373	0.022136	NA
114	10.532	0.014546	1.8373	0.022136	NA
115	10.532	0.014546	1.8373	0.022136	NA
116	10.532	0.014546	1.8373	0.022136	NA
119	10.532	0.014546	1.8373	0.022136	NA

120	10.532	0.014546	1.8373	0.022136	NA
121	10.532	0.014546	1.8373	0.022136	NA
123	10.532	0.014546	1.8373	0.022136	NA
127	10.532	0.014546	1.8373	0.022136	NA
128	10.532	0.014546	1.8373	0.022136	NA
2	10.421	0.015306	1.8151	0.022136	NA
13	10.421	0.015306	1.8151	0.022136	NA
67	10.421	0.015306	1.8151	0.022136	NA
5	10.385	0.015564	1.8079	0.022136	NA
7	10.385	0.015564	1.8079	0.022136	NA
9	10.385	0.015564	1.8079	0.022136	NA
11	10.385	0.015564	1.8079	0.022136	NA
12	10.385	0.015564	1.8079	0.022136	NA
18	10.385	0.015564	1.8079	0.022136	NA
19	10.385	0.015564	1.8079	0.022136	NA
20	10.385	0.015564	1.8079	0.022136	NA
21	10.385	0.015564	1.8079	0.022136	NA
23	10.385	0.015564	1.8079	0.022136	NA
27	10.385	0.015564	1.8079	0.022136	NA
30	10.385	0.015564	1.8079	0.022136	NA
32	10.385	0.015564	1.8079	0.022136	NA
33	10.385	0.015564	1.8079	0.022136	NA
34	10.385	0.015564	1.8079	0.022136	NA
35	10.385	0.015564	1.8079	0.022136	NA
36	10.385	0.015564	1.8079	0.022136	NA
53	10.385	0.015564	1.8079	0.022136	NA
60	10.385	0.015564	1.8079	0.022136	NA
61	10.385	0.015564	1.8079	0.022136	NA

65	10.385	0.015564	1.8079	0.022136	NA
73	10.385	0.015564	1.8079	0.022136	NA
80	10.385	0.015564	1.8079	0.022136	NA
86	10.385	0.015564	1.8079	0.022136	NA
90	10.385	0.015564	1.8079	0.022136	NA
91	10.385	0.015564	1.8079	0.022136	NA
100	10.385	0.015564	1.8079	0.022136	NA
102	10.385	0.015564	1.8079	0.022136	NA
104	10.385	0.015564	1.8079	0.022136	NA
126	10.385	0.015564	1.8079	0.022136	NA
117	10.116	0.017607	1.7543	0.024765	NA
16	9.9744	0.018785	1.7262	0.025855	NA
69	9.9744	0.018785	1.7262	0.025855	NA
1	9.8038	0.02031	1.6923	0.0262	NA
42	9.8038	0.02031	1.6923	0.0262	NA
49	9.8038	0.02031	1.6923	0.0262	NA
29	9.6667	0.021623	1.6651	0.0262	NA
38	9.6667	0.021623	1.6651	0.0262	NA
82	9.6667	0.021623	1.6651	0.0262	NA
14	9.5957	0.022334	1.651	0.0262	NA
40	9.5957	0.022334	1.651	0.0262	NA
45	9.5957	0.022334	1.651	0.0262	NA
57	9.5957	0.022334	1.651	0.0262	NA
58	9.5957	0.022334	1.651	0.0262	NA
77	9.5957	0.022334	1.651	0.0262	NA
99	9.5957	0.022334	1.651	0.0262	NA
125	9.5957	0.022334	1.651	0.0262	NA
129	9.5957	0.022334	1.651	0.0262	NA

124	9.5128	0.023195	1.6346	0.0262	NA
46	9.4917	0.02342	1.6304	0.0262	NA
109	9.4917	0.02342	1.6304	0.0262	NA
17	9.4615	0.023744	1.6244	0.0262	NA
22	9.4615	0.023744	1.6244	0.0262	NA
28	9.4615	0.023744	1.6244	0.0262	NA
31	9.4615	0.023744	1.6244	0.0262	NA
79	9.4615	0.023744	1.6244	0.0262	NA
25	9.359	0.02488	1.6042	0.026539	NA
37	9.359	0.02488	1.6042	0.026539	NA
62	9.359	0.02488	1.6042	0.026539	NA
93	9.359	0.02488	1.6042	0.026539	NA
81	8.8974	0.030686	1.5131	0.032461	NA
107	8.6923	0.033674	1.4727	0.03533	NA
105	8.5385	0.0361	1.4425	0.037568	NA
71	8.0769	0.044448	1.3521	0.045515	NA
122	8.0769	0.044448	1.3521	0.045515	NA
